# Metabolomics Profile of the Secretome of Space-Flown Oligodendrocytes

**DOI:** 10.3390/cells12182249

**Published:** 2023-09-11

**Authors:** Laurent Vergnes, Bernard Foucaud, Carlos Cepeda, Araceli Espinosa-Jeffrey

**Affiliations:** 1 Department of Human Genetics, David Geffen School of Medicine, University of California Los Angeles, Los Angeles, CA 90095, USA; lvergnes@g.ucla.edu; 2 Department of Psychiatry, Semel Institute for Neuroscience and Human Behavior, University of California Los Angeles, Los Angeles, CA 90095, USA; bernardfoucaud@sfr.fr (B.F.); ccepeda@ucla.edu (C.C.)

**Keywords:** microgravity, oligodendrocyte progenitors, global metabolomics, oligodendrocyte progenitor energetics

## Abstract

Intracranial hypertension (ICP) and visual impairment intracranial pressure (VIIP) are some of the sequels of long-term space missions. Here we sought to determine how space microgravity (µG) impacts the metabolomics profile of oligodendrocyte progenitors (OLPs), the myelin-forming cells in the central nervous system. We report increased glutamate and energy metabolism while the OLPs were in space for 26 days. We also show that after space flight, OLPs (SPC OLPs) display significantly increased mitochondrial respiration and glycolysis. These data are in agreement with our previous work using simulated microgravity. In addition, our global metabolomics approach allowed for the discovery of endogenous metabolites secreted by OLPs while in space that are significantly modulated by microgravity. Our results provide, for the first time, relevant information about the energetic state of OLPs while in space and after space flight. The functional and molecular relevance of these specific pathways are promising targets for therapeutic intervention for humans in long-term space missions to the moon, Mars and beyond.

## 1. Introduction

Numerous studies have shown that in normal mammalian cells grown in culture, microgravity induces an array of phenomena such as apoptosis and changes in their cytoskeleton, as well as in signal transduction, cell differentiation, proliferation, migration and adhesion. Effects differ depending on the cell type [1]. Microgravity also alters differentiation and augments apoptosis in thyroid carcinoma cells [2,3] and alters breast cancer-derived cells [4]. Microgravity is thus considered as a platform to study the metabolism of normal, developing, mature and cancerous cells, as well as the potential pharmacological approaches to treat, on Earth, neurodegeneration, developmental disabilities and cancer.

We have pioneered the study of simulated microgravity (sim-μG) using the “the 3D-clinostat” robot (Mitsubishi Heavy Industries) on neural cells and, in particular, neural stem cells and oligodendrocytes (OLs). OLs are the myelinating cells in the central nervous system (CNS) and are essential for the efficient synthesis and maintenance of myelin [5,6]. They form the compact segments of the myelin membrane that coil around the axon and allow for the saltatory conduction of action potentials, ensuring the healthy functioning of the CNS. Neurological diseases involving this cell type are characterized by myelin loss and an impairment in nerve conduction. A lack of or damaged myelin is debilitating and disabling because the nerve impulses slow down or even stop, causing neurological deficits. Understanding how OL progenitors (OLPs) differentiate and form myelin sheaths around nerve axons is of interest for the prevention and treatment of myelin-associated diseases. In many cases, OLPs fail to mature and, therefore, they cannot remyelinate axons in dys- and de-myelinating diseases such as multiple sclerosis (MS), Pelizaeus–Merzbacher disease or after a traumatic injury. Moreover, a chronic lack of myelin leads to axonal transection [7].

As neurological impairments have been observed after human space journeys, e.g., intracranial hypertension (ICP) and visual impairment intracranial pressure (VIIP), we have studied in previous papers the effects of artificial microgravity on cultured OLPs. Our first study using BrdU revealed that rodent and human OLPs displayed enhanced and sustained proliferation while they were in sim-µG with a concomitant shortening of the cell cycle. Moreover, these cells appeared to remember having been in µG and continued to proliferate more after having been placed in Earth’s gravity [8].

More recently, we studied the effects of space-µG on OLPs and found enhanced proliferation [9], yet some of them displayed incomplete cytokinesis [10]. Moreover, based on the expression of stage-specific developmental markers, we discovered that these cells came back in a more immature stage than that of the ground control OLPs [10]. Here we sought to determine A) the impact of space flight on the global metabolomic profile of the secretome produced by OLPs while in space-µG in order to start unraveling extracellular processes, as well as identifying gravitational-sensitive molecules, and B) the energetic status of OLPs after space flight. We found the presence of some gravitational-sensitive molecules, as well as enhanced energy and glutamate metabolism in the secretome generated while the OLPs were on board the International Space Station (ISS). Notably, we found an elevated energy demand in the OLPs upon return to Earth, which led to an increased use of glycolysis to support their energy demands.

In previous decades, we have studied and developed culture conditions for OLs and their precursors using biochemical, histochemical and morphological signals [11,12]. Key features of OLs are their well-defined developmental stages characterized by the expression of numerous stage-specific markers from which their expression is either transient or permanent throughout the life of these cells. These markers also define the functions that OLs support, including the production and secretion of the iron carrier glycoprotein transferrin for its autocrine and paracrine use in CNS iron metabolism, as well as the synthesis of all myelin components when OLs mature until they reach the myelinating stage. These stages and markers have been reported and reviewed by us and other scientists [5,13,14]. For the purpose of understanding the contents of the secretome produced by space-flown OLPs, here we use our previous description of these developmental stages [15,16] (Supplementary Figure S1).

## 2. Materials and Methods

### 2.1. Culture System for OLPs and Pre-Myelinating OLs

For the present study, we used human induced pluripotent (hiPS)-derived OLPs. Four years prior to the space flight, we established a culture system with a homogeneous population of NSCs that was obtained from the original hiPS cell line “CS83iCTR-33-n1” (fibroblasts), “reprogrammed” and provided to us by Cedars-Sinai Medical Center via a material transfer agreement. We then prepared embryoid bodies and subsequently converted them to “OLPs” using the protocol previously described by Espinosa-Jeffrey et al. [12]. For the purpose of this study, we used the oligodendrocyte specification medium (OSM), whose composition is shown in Table 1. This medium was used for pre-flight and during the experiment on board the ISS. The return medium is named BS1; to prepare BS1, we used OSM without N2 and B-27 and added Insulin Growth factor 1 (IGF-1) (Table 1).

### 2.2. Hardware and Space Flight

**Figure 1:**
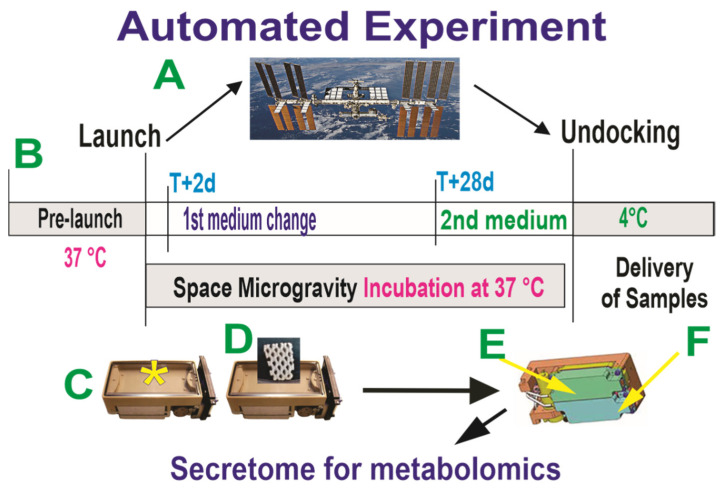
Collection system of secretome produced while cells were in space. Human OLPs, as part of the Bioscience-4 Space Biology NASA experiment, flew to the ISS on board SpaceX-21. This experiment was launched on 6 December 2020, and splashed down on 13 January 2021. These “active” experiments were designed to collect the conditioned medium (secretome) after the OLPs reached space and just before unberth to be able to capture the molecules secreted solely while cells were onboard the ISS (**A**). (**B**) The timeline depicting the handover moment and pre-launch. The travel culture medium was removed at T + 2 days and a fresh medium was left with the cells for 26 days, at which time the second culture medium was added to the cultures (a total of 26d + 2d) in space and the secretome was produced over 26 days while in space. (**C**) Cells travelled at 37 °C while in space for 28 days in the OSM contained in the cell chamber (asterisk). Cells were fed with BS1 on day T + 29 and returned to Earth in the same culture medium. The return medium was BS1 (see Table 1) from day 29 until delivery to the PI at UCLA. (**D**) View of the mesh carrier (2 mm × 2 mm). (**E**,**F**) Underneath the cell chamber, there are two tanks containing the fresh medium where the secretome was collected prior to unberth (arrows). This set of chambers was returned to Earth at 37 °C with a slow cool down in the second medium. The same number of chambers were flown to space or stayed on Earth in our laboratory as ground control. The OLPs travelled back from space in the second medium. Ground control OLPs were also seeded in these containers on mesh carriers and maintained in our laboratory at the same temperature, culture medium and conditions of space-flown OLPs. The only difference was Earth’s gravity vs. space microgravity. Upon splashdown, they were transferred from the Kennedy Space Center to the World Carrier and brought to our laboratory (UCLA) at 37 °C. (**F**) The mesh carriers populated with cells were transferred to poly-d-lysine=coated culture flasks and fed with a fresh medium. The secretome of each container and the tanks were all collected separately in numbered tubes and stored frozen at −80 °C. This medium is generally known as the conditioned medium. For the purpose of this manuscript, we refer to it as “secretome”.

OLPs grow optimally at a pH of 7.4; the culture medium was buffered with HEPES and its pH was adjusted to 7.4 for the space flight. The culture medium that fed the cells during the ascent to space was recovered automatically from the cell chamber. Subsequently, the fresh medium in tank 1 fed the OLPs while onboard the ISS for 26 days, as shown in Figure 1.

### 2.3. Secretome Metabolomics

Metabolic profiles were obtained for each individual group using the Metabolon Platform (Metabolon). Briefly, each sample or “group” consisted of 5 biological replicate specimens per group. Three groups of samples were compared: OSM 26D (*n* = 5), which consisted of the culture medium alone incubated in the same conditions and the hardware used for our cell cultures, 1G OLPs (*n* = 5) maintained in Earth gravity, and SPC OLPs (*n* = 5). Post-harvest, the conditioned medium was used to analyze, in a comparative manner, the molecules secreted by the OLPs in both conditions. This medium was subjected to methanol extraction, then split into aliquots for analysis via ultra-high-performance liquid chromatography/mass spectrometry (UHPLC/MS) in the positive (two methods), negative or polar ion mode. Metabolites were identified via the automated comparison of ion features to a reference library of chemical standards, followed by visual inspection for quality control, as previously described [17]. Compound Identification: Raw data was extracted, peak-identified and QC processed. Biochemical identifications are based on three criteria: retention index within a narrow RI window of the proposed identification, accurate mass match to the library +/− 10 ppm, and the MS/MS forward and reverse scores between the experimental data and authentic standards. The retention time/index (RI), mass to charge ratio (*m*/*z)*, and chromatographic data (including MS/MS spectral data) are used simultaneously to distinguish and differentiate biochemicals. The MS/MS scores are based on the comparison of ions present in the experimental spectrum to the ions present in the library spectrum. While there may be similarities between these molecules based on just one of these factors, the use of all three data points simultaneously, provides more accuracy for the identification of the biochemicals. The repertoire of “standards”, consisting of 3300 purified molecules, is available for this type of analysis.

For structurally unnamed biochemical, mass spectral entries have been created and identified by virtue of their recurrent nature (both chromatographic and mass spectral). The asterisk in figures indicates that this compound has been identified based on two of the three characteristics mentioned above. Compounds identified in this manner, have the potential for their future identification by classical structural analysis. Alternatively, a new standard may be added to the library and the biochemical name confirmed.

### 2.4. Statistical Analysis for the Metabolomics Study

To determine the statistical significance, Welsh’s two-sample *t*-tests were performed on the SPC, 1G and control medium groups of *n* = 5 in ArrayStudio (Omicsoft) or “R” to compare data between them; *p* < 0.05 was considered significant (Array Studio, RRID:SCR_010970). The false discovery rate (Q-value) was calculated to take into account the multiple comparisons that normally occur in metabolomic-based studies, with Q < 0.05 used as an indication of high confidence in a result. Principal Component Analysis (PCA) and Hierarchical Clustering Analysis (HCA) were performed in ArrayStudio. Random forest analysis was performed as described [18,19]. For the box plots, whiskers reflect the 5th and 95th percentiles (with the box displaying the 25th to 75th quartiles); the bisecting line represents the population median, while “+” represents the population mean, [20]. We only considered the molecules that presented a significant difference (*p* < 0.05) between the groups shown, with a metabolite ratio of <1.00.

### 2.5. Launch to the ISS

**Figure 2:**
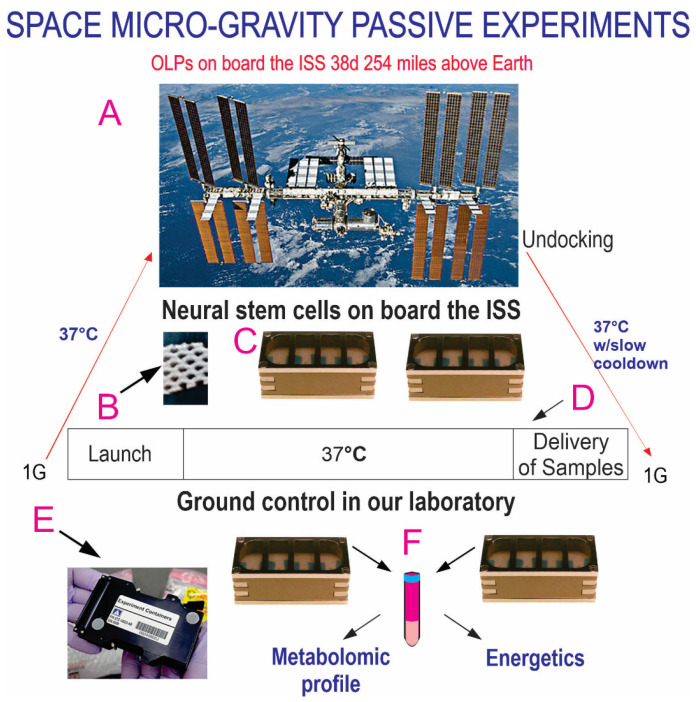
Synopsis of passive experiments. OLPs came back alive and were used for the respirometry analysis, a part of the Bioscience-4 space biology NASA experiment. These cells were flown to the ISS on board SpaceX-21 (**A**). It was launched on 6 December 2020 and landed (splashdown) on 13 January 2021. The cells remained in microgravity for 38 days. Its orbit height was 254 miles; its speed at orbit was 4.76 miles/s; and maximal speed reached 17,400 mph. On Earth, humans are exposed to 3 to 4 millisieverts (mSv) of radiation from natural sources per year, mainly from cosmic rays that make it through the atmosphere. On the ISS, astronauts receive about 150 mGv per six months. For the current mission, the average daily total radiation dose was 0.425 (mGv) on board the space station. This experiment is called “passive” because it was designed to mimic the trajectory that astronauts’ brains undergo during space flight (i.e., launch, stay in space, and splashdown when returning to Earth) without manipulation or medium change. (**B**) Example of mesh without cells. OLPs were seeded on the floating mesh carriers. (**C**) View of the 8-well Petri dishes used for the passive experiments on Earth. (**D**) All cells were maintained at 37 °C for the duration of the experiment. (**E**) View of the external shell in which the units travelled and stayed while in space. The same number of chambers was flown to space or stayed on Earth in our laboratory as ground control. Ground control cells were also seeded in these containers and maintained at the same temperature and conditions in our laboratory; the only difference was gravity vs. microgravity. (**F**) Secretome collected from each well separately. Upon splashdown, they were transferred from the Kennedy Space Center to the World Carrier and brought to our laboratory (UCLA) at 37 °C.

The Bioscience-4 mission launched onboard the Space-X 21 Dragon capsule on 6 December 2020. This is the first study to investigate the proliferation of human normal hiPS-derived OLPs in space microgravity. For space flight, the OLPs were seeded onto passive 8-well Petri dishes from Airbus–Kiwi (Friedrichshafen, Germany) on floating mesh carriers measuring 2 mm × 2 mm, to which the cells adhered firmly; this feature was necessary to ensure that the cells would not detach and die during launching or while returning to Earth. The OLPs were flown to the ISS and installed in the Space Technology and Advanced Research System Experiment Facility-1 (STAaRS F-1) at 37 °C. The cells remained onboard the ISS for 39 days and 9 h and then returned to Earth (Figure 2).

### 2.6. Cellular Bioenergetics

Upon return from space, we ascertained a drop of pH from 7.4 to 7.1, which should represent the negligible effect of CO2 levels on the cells’ metabolism. For the assay, the OLPs were seeded onto an XF24 Cell Culture Microplate (Agilent Technologies, Santa Clara, CA, USA) at 5 × 10^4^ cells per well in OSM. The cells were kept for 24 h in standard culture conditions (37 °C, 4.5% CO_2_). The following day, the wells were filled with fresh OSM. The following day, some of the supernatant was removed from the well plates. We used an XF24 Analyzer (Seahorse Bioscience, North Billerica, MA, USA) to measure the oxygen consumption rate (OCR) and extracellular acidification rate (ECAR). The OCR and ECAR reflect the rates of cellular respiration and glycolysis, respectively. Measurements were performed with the cells in unbuffered Dulbecco’s Modified Eagle Medium (from Sigma-Aldrich, St. Louis, MO, USA) supplemented with 1 mM of pyruvate, 2 mM of glutamine, and 25 mM of glucose. Measures were recorded before (basal reading) and after the sequential injections of oligomycin, FCCP and rotenone/myxothiazol. The mitochondrial respiration was the difference between the total and rotenone/myxothiazol response. The maximal OCR was the response to the FCCP, whereas the maximal ECAR was the response to oligomycin. Values were normalized to the cellular protein concentration, determined with the protein assay reagent (Bio-Rad, Hercules, CA, USA) in all experiments.

## 3. Results

### 3.1. Global Profile of the Secretome from the Different Treatments

**Figure 3:**
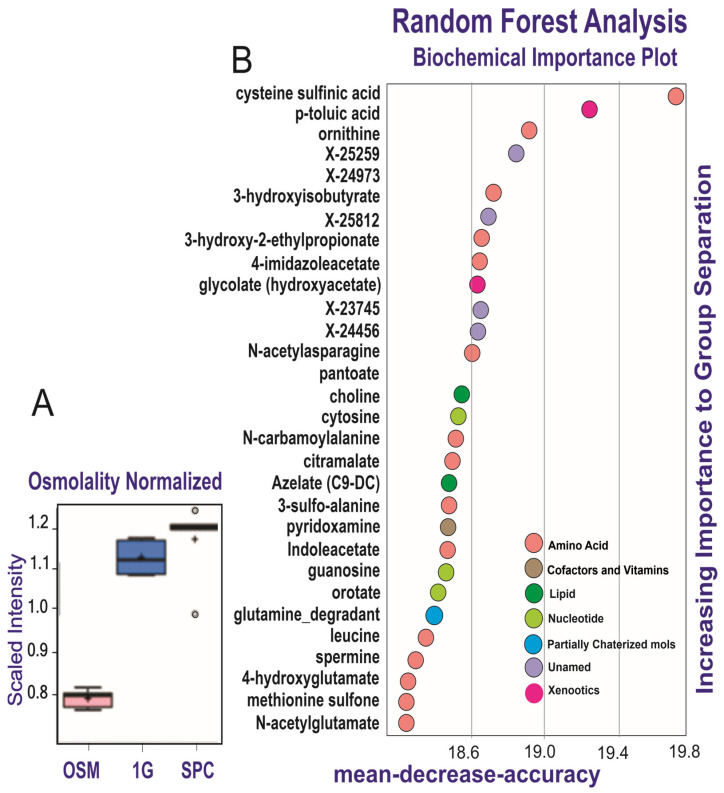
Discriminative power of secretome analysis. The diagram (**A**) shows the comparison of metabolites secreted by OLPs with regard to their media and SPC vs. 1G. The random forest analysis (**B**) represents the 30 top-ranking biochemicals in the secretomes.

Secretomes of the OLPs kept for 26 days in 1G (1G OLP 26D) or SPC (SPC OLP 26D) were compared to the reference medium (OSM alone), using five biological replicates (see Section 2). Their mass spectra were globally examined, then compared one by one for the intensity of their components’ signals, to evidence a specific profile for each of these groups. Altogether, 424 signals were recorded, among which 372 were identified. Table 2 compares in a global way the mass spectra of the two groups of secretomes to those of the OSM alone. They display differences in the number of signals whose intensity is modified. There were more metabolites that increased in the presence of cells (157 and 182 in 1G and SPC, respectively; *p* < 0.05) compared to decreased metabolites (90 and 55 in 1G and SPC, respectively; *p* < 0.05). Importantly, there were 136 metabolites that increased and 62 metabolites that decreased in SPC vs. 1G. Thus, after 26 days, an increase was observed for ca. 60% of the signals present in the secretome of the cells kept in 1G, while this increase was of ca. 40% in space; when these secretomes are compared to the OSM, a larger number of metabolites was secreted at normal gravity than in its absence. The osmolality box plot represents the cumulative signal intensities in cell secretomes. The osmolality was higher in the presence of cells than in the media alone, and was increased in the cells at 0G compared to 1G (Figure 3A). Altogether, these results indicate a quantitative and qualitative variation in the secretome of cells at 0G compared to 1G. These changes in intensity do not equally affect all signals: the plot in Figure 3B (random forest analysis) shows the top metabolites (notably amino acids and xenobiotics) differentiating the OSM 26D medium from the secretomes, either in 1G or SPC conditions.

### 3.2. Glycolysis and Energy-Related Metabolism

**Figure 4:**
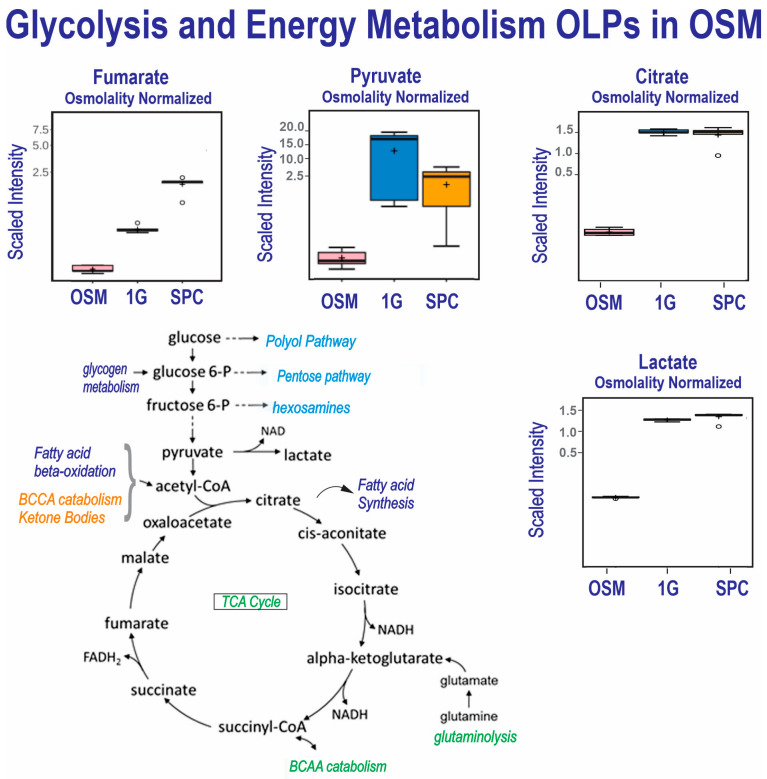
Comparison of metabolite utilization by 0G and 1G OLPs showed that in space, OLPs secreted significantly more fumarate than their 1G counterparts. OLPs from both groups secreted high levels of citrate and lactate, although with a slight but significantly higher level in 0G OLPs. (*n* = 5). For box plots, whiskers reflect 5th and 95th percentiles (with the box displaying 25th to 75th quartiles); bisecting line represents the population median, while the plus sign (+) represents population mean; for details, see [21].

The comparison of the control medium and the secretomes from the OLPs grown in SPC or 1G shows that the SPC OLPs secreted about half of the pyruvate and phosphates of the 1G OLPs, suggesting the high energy requirements of OLPs in space. Both the SPC and 1G secretomes contained elevated levels of lactate, citrate, aconitate and malate vs. OSM but at levels between 1G and SPC, indicating a comparable metabolism in the first part of the TCA cycle. In contrast, alpha-ketoglutarate, succinate, fumarate and malate displayed increased levels in both the SPC and 1G secretomes, but with a larger increase in SPC, suggesting a change in glutaminolysis and/or a BCCA catabolism influx in SPC compared to 1G (see TCA cycle, Figure 4. The scaled intensity figures illustrate how the 1G and SPC secretomes differ for their contents in pyruvate and fumarate. Pyruvate (a product of the glutamate pathway) is one of the main sources of energy for respiration and energy production and it was decreased in the OLPs flown to space. It fuels the TCA cycle (glycolysis pathway scheme), which generates energy (stored as phosphorylated and reduced coenzymes), while fumarate and malate are end products in this cycle (Table 3 and Figure 4), as already reported [16].

### 3.3. Differences in Glutamate Metabolism

**Figure 5:**
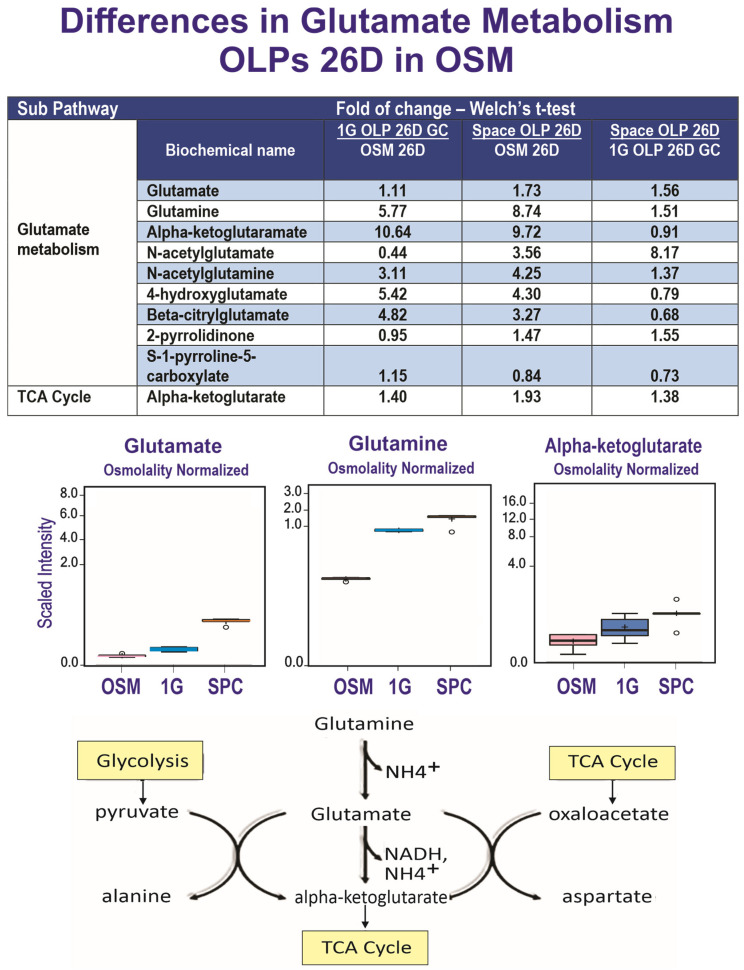
Comparison of metabolite utilization by SPC and 1G OLPs showed that in space, OLPs secreted significantly more glutamate and glutamine while both groups secreted similar levels of alpha-ketoglutarate (1G) (*n* = 5). For box plots, whiskers reflect 5th and 95th percentiles (with the box displaying 25th to 75th quartiles); bisecting line represents the population median, while the plus sign (+) represents population mean; for details, see [20].

The detected metabolites were classified by their metabolic identity. Our present data confirm that OLs secrete glutamate metabolites into their milieu: α-ketoglutarate (α-KG), (1G), glutamine, and other glutamate derivatives were released at a high level in both 1G and SPC conditions vs. OSM; among those, the release was 40 to 55% higher in space for glutamine, glutamate and α-KG, while 4-hydroxy- and ß-citryl-glutamate derivatives were lower (20–30%). N-acetyl-glutamate was lower in the 1G medium than in OSM but higher in SPC vs. OSM, suggesting a shift from uptake to secretion in 1G to SPC. The scaled intensity block diagrams for glutamate, glutamine and α-KG illustrate that the three of them are released by the cells, and that this release is accentuated by SPC (Figure 5).

### 3.4. Differences in Lipid Metabolism

**Figure 6:**
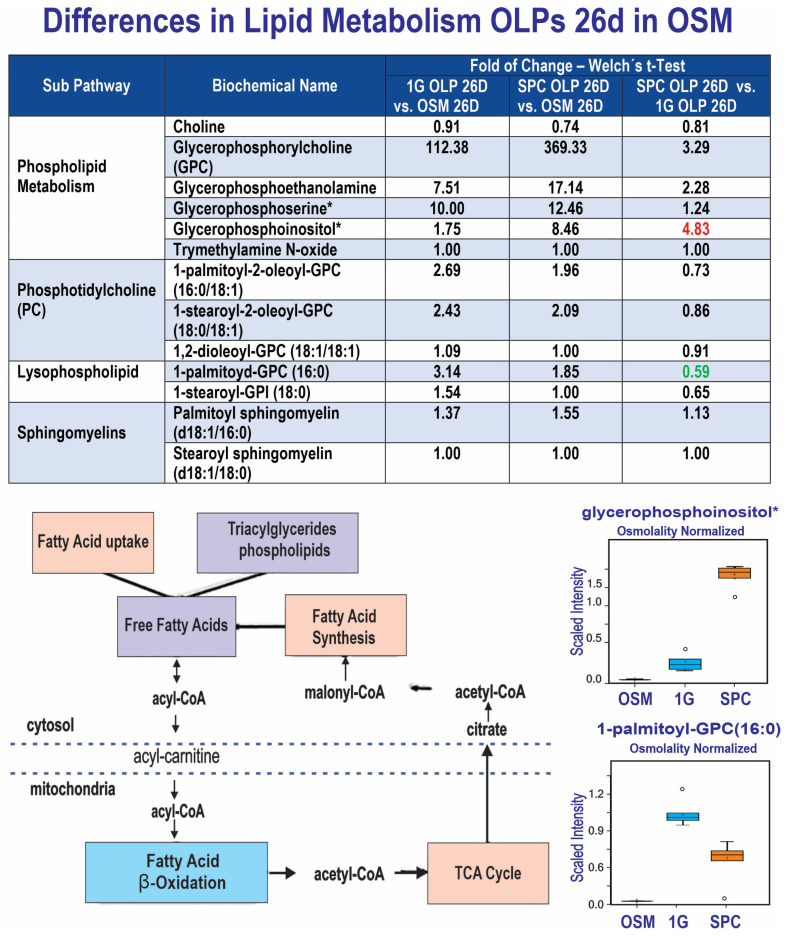
Comparison of metabolite utilization by SPC and 1G OLPs showed that in space, OLPs secreted significantly more glycerophosphoinositol than OLPs in 1G. In contrast, SPC OLPs secreted less palmitoyl-GPC than 1G OLPs (*n* = 5). The asterisk indicates that this compound was identified based on two of the three characteristics mentioned in Methods. Compounds identified in this manner, have the potential for their future identification by classical structural analysis. Alternatively, a new standard may be added to the library and the biochemical name confirmed. For box plots, whiskers reflect 5th and 95th percentiles (with the box displaying 25th to 75th quartiles); bisecting line represents the population median, while the plus sign (+) represents population mean.

Glycerophosphate lipids (phospholipids) generated higher signals in the cell secretomes and were more secreted by SPC cells vs. 1G cells, consistent with previous observations [16]. However, phosphatidyl-cholines with short chains of unsaturated fatty acids and lysophospholipids (non myelin-specific species) were also more detected in cell secretome, but at a lower level in SPC than 1G. In the case of sphingomyelins, their levels of secretion were similar between the 1G and SPC OLPs. Taken together, these observations indicate that our OLPs have their developmental program at the very least delayed in the absence of gravity (Figure 6).

### 3.5. Space Flight Elicits Higher Respiration Rates in hiPS-Derived OLPs

**Figure 7:**
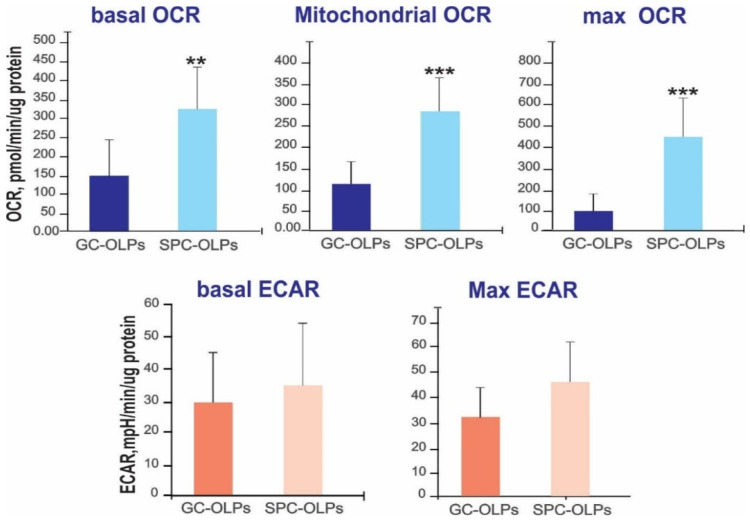
Space-flown OLPs and 1G cells were cultured in Seahorse Bioscience V7 plate for 20 h. A higher basal OCR, mitochondrial and higher maximal OCR showed significant differences with regard to SPC OLPs, indicating that the cells were in a higher bioenergetics state. Interestingly, there were not significant differences for the ECAR. There were 12 samples per experiment. Values are expressed as mean + SD. Student’s *t*-test revealed that these differences were **: *p* < 0.01 ***: *p* < 0.001 vs. respective controls.

In order to ascertain the effects of space-µG on energy metabolism, we compared 1G OLPs and SPC OLPs. We quantified the OCR and ECAR as measures of mitochondrial respiration and glycolysis, respectively. We found that five days after splashdown, the SPC OLPs presented significantly higher basal, mitochondrial and maximal respiration when compared with the 1G OLPs, suggesting a higher mitochondria-dependent energy demand. In contrast, the glycolysis differences were not statistically significant (Figure 7).

## 4. Discussion

We all were born, developed and have lived in Earth’s gravity and, for technical reasons, the effect of gravity has been, so far, poorly explored, although its mechanical effects are germane to the well-documented effect of cell contacts during their growth, development and differentiation. In our current study, we examined the metabolomic profile of the secretome produced by OLPs in space and discovered subtle but significant changes in the metabolite levels of the SPC OLPs vs. the 1G OLPs. Indeed, we found a differential regulation of metabolites in response to weightlessness. In addition, glutamate production and energy requirements were also increased.

### 4.1. Glycolysis and Energy Related Metabolites

The total energy intake of the brain corresponds to 20% of overall energy intake, even though it weighs 2% of a person’s weight. During development, glucose and lactate are the main source of energy for oligodendrocyte progenitors (OLPs) [21]. OLs use lactate as a source of energy as well as a precursor for lipid synthesis by converting it to pyruvate. Subsequently, pyruvate is transported to the mitochondria for the tricarboxylic acid cycle (TCA), or converted to ATP in their cytosol via glycolysis. During development and myelinogenesis, OLs have high energy demands and are extremely vulnerable to energy deprivation [22]. Metabolic dysfunction is commonly associated with MS [23,24]. In these patients, acute hypoxia-like lesions, mitochondrial impairment and a reduction in complex I and complex IV of the electron transport chain are reduced [25]. There are several patterns of de-myelination and several classifications of MS. One is named oligodendrogliopathy, where the cell processes of OLs are dying back, and it is the distinctive characteristic for “pattern III” MS [26]. It has been reported that OLs’ metabolism is impacted by dimethyl-fumarate (DMF, Tecfidera^®^). This compound is being used for the treatment of MS [27,28]. It is believed that it modulates both antioxidant and lipid metabolism in OLs [29]. Patients treated with this molecule displayed a higher brain magnetization transfer ratio (MTR), and this was thought to correlate with the preservation of myelin density [30]. As observed previously in sim-μG, the OLPs secreted more fumarate and methyl-fumarate (mesaconate) in SPC while maintaining their progenitor stage [12]. Here, we show that the OLPs exposed to space μG displayed an enhanced need for energy with regard to their ground control counterparts. In addition, more lactate (produced during anaerobic oxidation to fulfill energy needs) was also significantly released by the SPC OLPs. The use of extra energy in the SPC OLPs cannot be explained through a stimulation of myelin synthesis; glycerate, a building element in the formation of triglycerides, was secreted by these cells, indicating that they were still at the immature developmental stage, while the 1G OLPs may have been moving forward towards maturation as previously demonstrated by Tran et al. [10]. Further studies are needed to ascertain the causes of such enhanced energy consumption by OLPs.

### 4.2. Glutamate Metabolism

The glutamate pathway is a hub: glutamate forms from glutamine via deamination through phosphate-activated glutaminase; it is fed by the glycolysis (with alanine as a by-product); glutamate is also a co-substrate in all reactions catalyzed by aminotransferases, which convert it to α-KG, a key intermediate of the TCA cycle [31]; glutamate generates energy itself (NADH). Astrocytes are thought to be the only suppliers to the glutamate metabolism in the brain [32]. Nonetheless, it has been shown that OLs also express high levels of glutamate synthetase (GS) at the level of the midbrain and the spinal cord [33]. These authors also reported that OLs do not need glutamate for their survival or for their myelinating function, thus indicating that its synthesis in OLs is part of their genetic blueprint, where no neuronal signal is required. GS also catalyzes the metabolization of glutamate into glutamine [33]. But the most important fact is that the selective lack of glutamine and glutamate in the brain results in the neonate’s death [34], and the deletion of glutamate in OLs results in the disruption of neuronal (synaptic) glutamate signaling that consequently alters glutamate-dependent behavior [33]. Our data appear to complement those of the above-mentioned authors: they report that GS expression occurs “late” in their development. Here, we demonstrate that OLs express glutamate at an early, pre-myelinating stage, and OLPs in space secrete more glutamate than in 1G. Therefore, our novel data indicate that not only mature OLs support glutamatergic transmission through GS, but their progenitors may also play an important role in the glutamatergic transmission pathway even before myelination takes place. In another model of cell differentiation, α-KG is shown to induce the demethylation of histones as a cofactor of histone demethylases [35] and hence influence the differentiation of the cells [36]. Glutamine, on the other hand, promotes pluripotent cells via the removal of differentiated cells in culture [37]. The pivotal role of the glutamate–glutamine pair in cell lineage specification and developmental stage maintenance at the OLP stage points to its involvement in the effects of reduced gravity on maintaining them at a progenitor stage, as previously shown by the expression of early OLP markers by our cells flown to space with regard to the 1G OLPs [10]. Glutamate dysregulation has been linked to numerous pathologies such as epilepsy and neurodegenerative diseases. Moreover, glutamate is present in sera from MS patients at higher levels than in the age-matched control population [38,39]. In addition, glutamate levels increase in the cerebrospinal fluid (CSF) [40] and the brains of MS patients [41,42]. Moreover, α-amino-3-hydroxy-5-methyl-4-isoxazolepropionic acid (AMPA), *N*-methyl-D-aspartate (NMDA) and kainate receptors are also upregulated [43]. Our data show a significant increase in glutamate in the secretome of OLPs flown into space; considering that in MS, OLPs are available around the lesion but they do not mature, it is possible that glutamate is made and secreted by those OLPs. Based on our above-mentioned data, where the SPC OLPs produced more glutamate than those kept in 1G, serum measurements of glutamate levels before, during and after space missions are of the essence to monitor changes produced by space microgravity in astronauts. This is important because the excessive stimulation of neurons by glutamate that can be caused by energy deficiency, oxidative stress, mitochondrial dysfunction and calcium overload leads to an excitotoxicity that is characterized by the exacerbation of glutamate receptors, triggering a cascade of toxic effects that leads to neuronal cell death [44,45] and, consequently, nearly all brain functions, including learning and memory, are affected.

### 4.3. Lipid Metabolism

The knowledge we have on the primary sources of energy for mammalian cells derives solely from experiments performed within Earth’s gravity. These sources are proteins, fats and sugars. Besides being a source of energy, lipids act as signaling molecules to direct OLPs and pre-myelinating OLs to either proliferation or maturation into myelinating OLs [46]. Moreover, fatty acids (FAS) are necessary for myelin synthesis. Myelin is the lipid-rich membrane that insulates the axons in the CNS, and it is essential for our daily life activities. Its composition differs from cellular membranes [47,48] in terms of their lipid to protein ratio, where lipids account for 70% myelin, whereas cholesterol and glycosphingolipids such as galactosylceramides, sulfatides and gangliosides account for 40% and 20% of the total lipids, respectively. Abnormal or impaired OLs result in severe CNS pathologies during development and in neurodegenerative disorders. In most myelin disorders, OLPs are present but fail to mature [49]. In the present study, we found that OLPs secreted phospholipids in a higher quantity in SPC than in 1G, except for certain species of phosphatidylcholine. In contrast, we did not observe differences in sphingomyelin secretion between 1G and SPC.

### 4.4. Post-Flight Energetics

Here, using respirometry measurements, we demonstrate important differences in energy metabolism. The SPC OLPs consumed more oxygen (basal respiration) and were able to increase respiration under stress (maximal respiration), suggesting a higher mitochondria-dependent energy demand, and probably a higher mitochondria content. Based on our previous work, we hypothesized that we would have more OLPs after space flight, yet we had to obtain confirmatory evidence. Both findings, the increased proliferation [8] and enhanced energy metabolism, are in agreement with our previous report where, using sim-µG, we revealed that there was increased mitochondrial respiration and increased glycolysis after 24 h of exposure to sim-µG [16]. Interestingly, in the case of the SPC OLPs, they showed preferential oxidative respiration rather than glycolysis. This is a different outcome from that obtained when using the 3D-clinostat. The reasons for these differences are currently unknown. Nonetheless, although the glycolytic reserve capacity appears not to have been used, the SPC OLPs still had higher energy demands after space flight.

We previously performed the same secretome analysis of OLPs in sim-µG [9,10]. Although there was major difference in the measure of the secretome of OLPs, like time (3 days vs. 26 days) and method (µG in 3D-clinostat vs. SPC), it is interesting to compare the similarities and the differences. In both instances, lipid secretion, especially phospholipids, was increased in µG/SPC. Mitochondrial respiration was also elevated in µG/SPC, suggesting that this increased ATP demand could be necessary to sustain lipid synthesis/secretion, as well as cell proliferation. Comparisons of the TCA cycle-related metabolites were not identical: more fumarate was secreted in µG/SPC, but the α-KG secretion was discordant [12].

### 4.5. General Considerations

We do not know how long human OLs would take to synthetize myelin and myelinate axons in space, but we recently showed that these cells expressed earlier OLP markers with regard to the 1G OLPs; thus, the maturation of these cells appeared to be delayed [10]. Here, we observe another indication of a delayed lineage progression. What we do not know is whether this delay is transient as part of adapting to weightlessness, or it would result in an arrest of their development. Longer studies with OLPs in space could answer these questions. Finally, the Seahorse experiments revealed significantly higher mitochondrial respiration in OLPs after space flight, revealing that OLPs and their progenies remember having been in space. This is a very important point, as the SPC OLPs also proliferated more in 1G after returning to Earth, pointing toward the imminent need to perform epigenetic studies. An important difference with our studies in sim-µG is that the glycolysis was equivalent in the SPC OLPs and 1G OLPs, indicating that they did not need to switch to glycolysis as their last resource for energy.

### 4.6. Significance

Since, in most myelin disorders, OLPs are present but fail to mature, our current study shows that glutamate and energy metabolism are significantly enhanced in response to microgravity. Therefore, both pathways are plausible targets to address OLPs’ maturation in MS, as well as other related myelin disorders and developmental disabilities like peri-ventricular leukomalacia, in order to enhance myelination in myelin-deficient CNSs.

## 5. Conclusions

We have started to unravel the effects of microgravity on the metabolic pathways inherent to OLPs that show significant changes. Nonetheless, so far, they appear not to be deleterious and appear to remain functional in astronauts. To the best of our knowledge, this is the first study that uses space as a novel platform to understand molecular changes in OLs at their progenitor stage. How this new knowledge is applicable to astronauts will become clearer when more studies of this nature are performed as they embark on long-term space missions.

Years ago, we started our work using microgravity to test if we could produce healthier human OLPs in short periods of time, aiming at transplanting them to remyelinate myelin-deficient CNSs, like in the case of MS or leukodystrophies. We have shown that both sim-µG and space microgravity increase OLPs’ proliferation. The present study provides evidence that OLPs produce glutamate and that this is enhanced by space flight, contributing perhaps to maintaining OLPs in an immature stage. This point is crucial to elucidate, as the modulation of glutamate in MS patients might lead to enhanced myelination.

## Figures and Tables

**Table 1:** Complete DMEM/F12 medium with supplements.

Reagents	OSM	BS1
Insulin	10 µg/mL	10 µg/mL
Transferrin	100 µg/mL	100 µg/mL
Putrescine	32.2 µg/mL	32.2 µg/mL
Na-bicarbonate	4.4 µg/mL	4.4 µg/mL
D(+) galactose	9.2 mg/mL	9.2 mg/mL
Kanamycin	16 ng/mL	16 ng/mL
Na-selenite	6.4 ng/mL	6.4 ng/mL
N2	2%	0
B-27	2%	0
IGF-1		160 ng/µL

**Table 2:** Comparative view of the number of metabolites that contribute to the secretome composition in SPC OLPs vs. 1G OLPs and vs. medium alone, respectively. Column one shows the secreted molecules produced by OLPs in Earth’s gravity. Column two shows the secreted molecules produced by OLPs over 26D while in the ISS (secretome from the tank found underneath the automated hardware units). Column three shows the difference of metabolites up or downregulated in SPC vs. 1G OLP secretome.

Number of Metabolites Measured in the Secretome’s Sample	1G OLP 26 D vs. OSM 26 D	SPC OLP 26 D vs. OSM 26 D	SPC OLP 26 D vs. 1G OLP 26 D
Total number of metabolites measured (secretome or medium)	247	237	198
Number of metabolites higher in the secretome than in the medium	157	182	136
Number of metabolites lower in the secretome than in the medium	90	55	62
Number of metabolites that decreased in the secretome, % of total	36	23	31

**Table 3:** Comparison of metabolites secreted by OLPs grown in 0G or 1G vs. OSM culture medium alone. The figures in columns 1 and 2 represent the ratio of the signal intensities between the secretomes (1 G or 0 G) and OSM. Column 3 compares these two ratios. The numbers in green indicate a low release (pyruvate, phosphate) and those in red a high release. The statistical significance of these ratios was rated applying the Welsh two-sample *t*-test, with rho ≤ 0.05 for the first nine metabolites and for phosphate, and ≤0.05 < rho < 0.1 for the others.

Sub Pathway	Biochemical Name	1 G OLP 26 D (GC)/ OSM 26 D	SPACE OLP 26 D/ OSM 26 D	SPACE OLP 26 D/ 1 G OLP 26 D (GC)
Glycolysis, Glyconeogenesis and pyruvate metabolism	Pyruvate	17.56	7.1	0.4
	Lactate	28.71	32.84	1.14
	Glycerate	0.49	0.96	1.97
TCA Cycle	Citrate	7.55	7.06	0.94
	Aconitate (cis or trans)	13.78	14.06	1.02
	Alppha keto-glutarate	1.40	1.93	1.38
	Succinate	2.45	4.07	1.66
	Fumarate	2.8	8.84	3.16
	Malate	3.81	4.69	1.23
	Itaconate	1.0	1.65	1.24
	Tricarballylate	0.96	1.06	1.11
	2-Methylcitrate/Honocitrate	0.73	1.13	1.44
	Mesaconate	0.96	2.0	2.09
	Citraconate/gluconate	0.88	2.02	2.31
Oxydative Phosphorylation	Phosphate	3.59	1.3	0.36

## Data Availability

All data are available from the PI upon reasonable request. All data obtained from this study have been included in the manuscript.

## Supplementary Materials

The following supporting information can be downloaded at: https://www.mdpi.com/article/10.3390/cells12182249/s1, Figure S1: OLs undergo sequential morphological changes starting from oligodendreoblasts (OLPs); Figure S2: Comparison of metabolite utilization by 0G and 1G OLPs showed that in space OLPs secreted significantly more mesaconate than their 1G counterparts.

## Abbreviations

ICP	Intracranial hypertension
VIIP	Visual impairment intracranial pressure
µG	Microgravity
OLPs	Oligodendrocyte progenitors
SPC OLPs	Space flight oligodendrocyte progenitors
sim-µG	Simulated microgravity
OLs	Oligodendrocytes
CNS	Central nervous system
MS	Multiple sclerosis
BrdU	Bromodeoxyuridine
ISS	International Space Station
hiPS	Human induced pluripotent stem cells
CS83iCTR-33-n1	Fibroblasts
N2	A supplement containing recombinant human insulin, human transferrin sodium selenite, putrescine and progesterone
B-27	Trademark
OSM	Oligodendrocyte specification medium
IGF-1	Insulin Growth Factor 1
DMEM/F12	Dulbecco’s Modified Eagle Medium/Nutrient Mixture F-12
*n* = 5	Five biological replicate specimens per group
1G	Earth Gravity
UHPLC/MS	Ultra-High-Performance Liquid Chromatography/Mass Spectrometry
Q-value	False discovery rate
PCA	Principal Component Analysis
HCA	Hierarchical Clustering Analysis
STAaRS	Space Technology and Advanced Research System Experiment Facility 1
NASA	National Aeronautics and Space Administration
mSv	Millisieverts
mGy	Milligray
OCR	Oxygen consumption rate
ECAR	Extracellular acidification rate
mM	Millimolar
1G OLP 26D	Earth gravity Oligodendrocyte Progenitors kept for 26 days
SPC OLP 26D	Space flight Oligodendrocyte Progenitors kept for 26 days
OSM 26D	Oligodendrocyte specification medium kept for 26 days
TCA Cycle	Tricarboxylic Acid Cycle
BCAA	Branched-Chain Amino Acid
NAD	Nicotinamide adenine dinucleotide
FADH_2_	Flavin adenine dinucleotide
α-KG	α-Ketoglutarate
NH_4_	Ammonium
ATP	Adenosine tri-phosphate
DMF	Dimethyl-fumarate
MTR	Magnetization Transfer Ratio
NADH	Nicotinamide adenine dinucleotide (NAD) + hydrogen (H)
GS	Glutamate Synthetase
CSF	Cerebrospinal fluid
AMPA	α-amino-3-hydroxy-5-methyl-4-isoxazolepropionic acid
NMDA	N-methyl-D-aspartate
FAS	Fatty acids
MTA	Material transfer agreement
