# Supplementary material for: Metabolomics Profile of the Secretome of Space-Flown Oligodendrocytes
Source: Cells. 2023 Sep 11;12(18):2249. doi: 10.3390/cells12182249 (PMC10528075; doi:10.3390/cells12182249)
Supplement: Supplementary file 1 [file cells-12-02249-s001.zip › cells-2594810-supplementary.pdf]

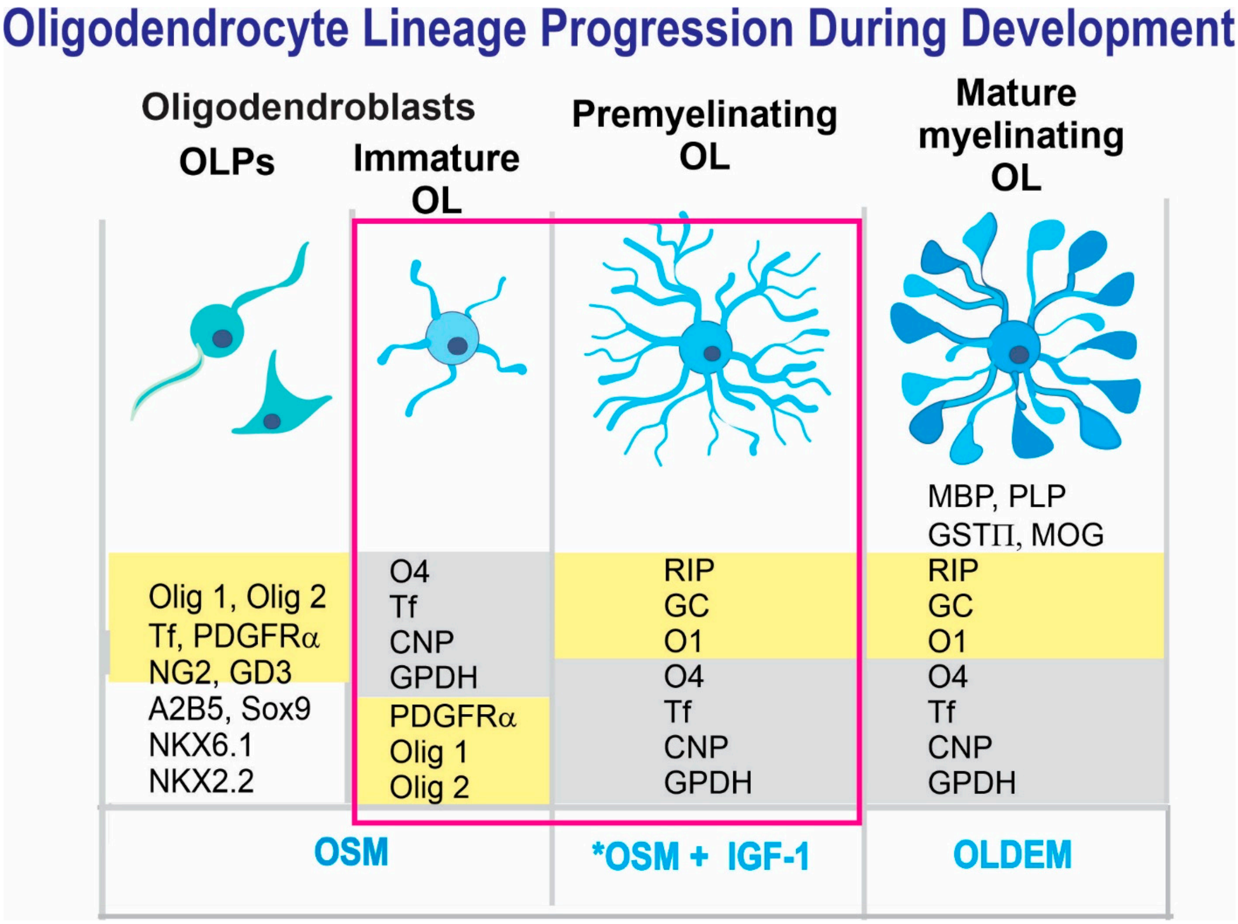

Figure S1. OLs undergo sequential morphological changes starting from oligodendreoblasts (OLPs). They acquire characteristics inherent to a functional OL when they mature. The partial list of markers below each developmental stage is not exhaustive, it rather represents frequently used markers to identify OLs and their specific developmental stage. The culture media mentioned above at the bottom of the specific stage, supports proliferation) and it also maintains the cells at this OLP stage. [11, 12]. Our OLPs travelled to space in OSM (oligodendrocyte specification medium), which was replaced with fresh medium 48h after arrival to the ISS. The secretome produced during 26D was collected prior to unberth.

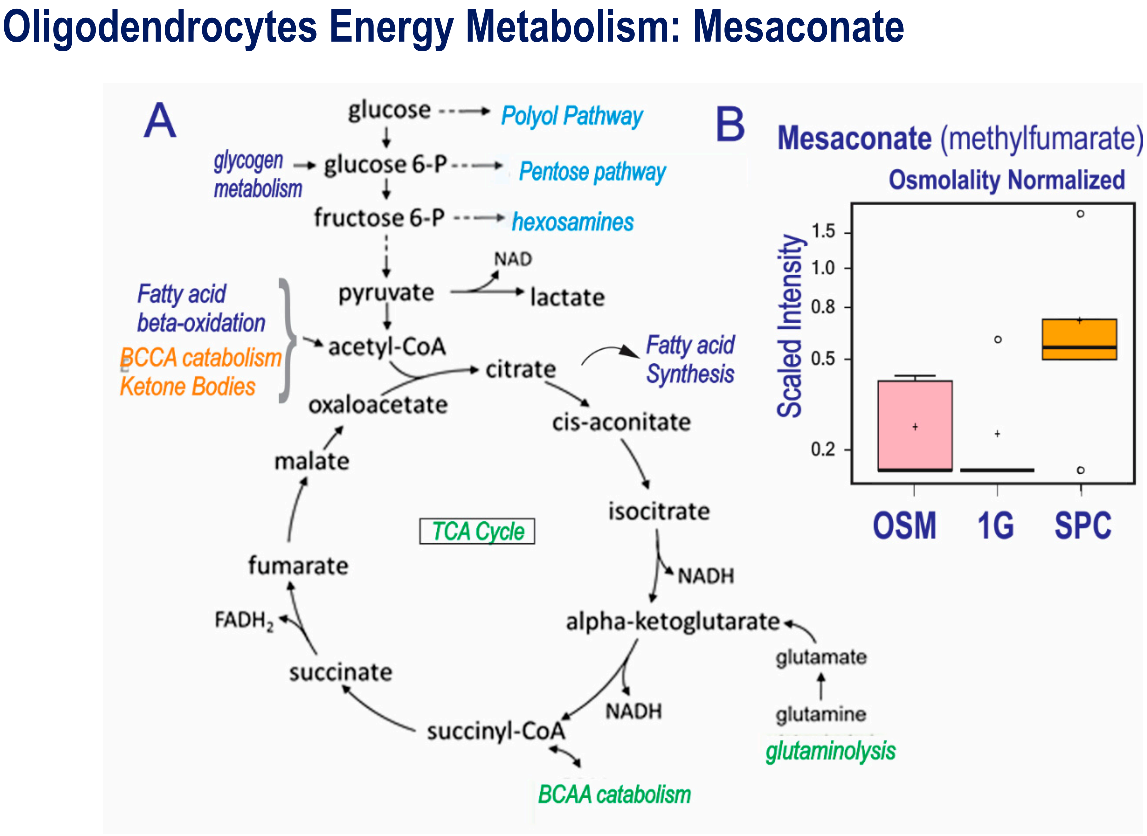

Figure S2. Comparison of metabolite utilization by 0G and 1G OLPs showed that in space OLPs secreted significantly more mesaconate than their 1G counterparts. For box plots, whiskers reflect 5th and 95th percentiles (with the box displaying 25th to 75th quartiles); bisecting line represents the population median, while the plus sign (+) represents population mean; for details, see [22].
